# AutonoMS: Automated Ion Mobility Metabolomic Fingerprinting

**DOI:** 10.1021/jasms.3c00396

**Published:** 2024-02-04

**Authors:** Gabriel K. Reder, Erik Y. Bjurström, Daniel Brunnsåker, Filip Kronström, Praphapan Lasin, Ievgeniia Tiukova, Otto I. Savolainen, James N. Dodds, Jody C. May, John P. Wikswo, John A. McLean, Ross D. King

**Affiliations:** † Department of Life Sciences, Chalmers University of Technology, Gothenburg 412 96, Sweden; ‡ Department of Computer Science and Engineering, Chalmers University of Technology, Gothenburg 412 96, Sweden; § Department of Applied Physics, SciLifeLab, KTH Royal Institute of Technology, Solna 171 21, Sweden; ∥ Institute of Public Health and Clinical Nutrition, University of Eastern Finland, Kuopio 702 11, Finland; ⊥ Chemistry Department, The University of North Carolina at Chapel Hill, Chapel Hill, North Carolina 27599, United States; # Department of Chemistry, Vanderbilt University, Nashville, Tennessee 37235, United States; ¶ Center for Innovative Technology, Vanderbilt University, Nashville, Tennessee 37235, United States; ∇ Vanderbilt Institute for Integrative Biosystems Research and Education, Vanderbilt University, Nashville, Tennessee 37235, United States; ○ Department of Biomedical Engineering, Vanderbilt University, Nashville, Tennessee 37235, United States; × Department of Physics and Astronomy, Vanderbilt University, Nashville, Tennessee 37235, United States; △ Department of Molecular Physiology and Biophysics, Vanderbilt University, Nashville, Tennessee 37240, United States; ● Department of Chemical Engineering and Biotechnology, University of Cambridge, Cambridge CB3 0AS, U.K.; ∞ The Alan Turing Institute, London NW1 2DB, U.K.

## Abstract

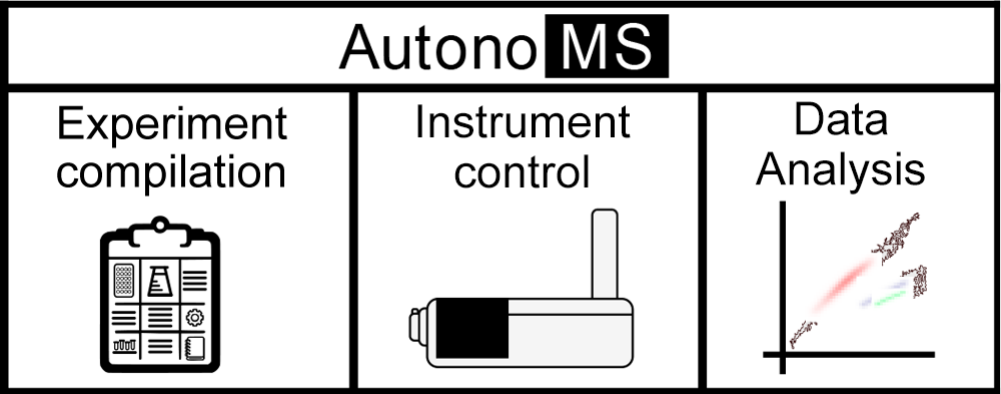

Automation is dramatically changing the nature of laboratory life science. Robotic lab hardware that can perform manual operations with greater speed, endurance, and reproducibility opens an avenue for faster scientific discovery with less time spent on laborious repetitive tasks. A major bottleneck remains in integrating cutting-edge laboratory equipment into automated workflows, notably specialized analytical equipment, which is designed for human usage. Here we present AutonoMS, a platform for automatically running, processing, and analyzing high-throughput mass spectrometry experiments. AutonoMS is currently written around an ion mobility mass spectrometry (IM-MS) platform and can be adapted to additional analytical instruments and data processing flows. AutonoMS enables automated software agent-controlled end-to-end measurement and analysis runs from experimental specification files that can be produced by human users or upstream software processes. We demonstrate the use and abilities of AutonoMS in a high-throughput flow-injection ion mobility configuration with 5 s sample analysis time, processing robotically prepared chemical standards and cultured yeast samples in targeted and untargeted metabolomics applications. The platform exhibited consistency, reliability, and ease of use while eliminating the need for human intervention in the process of sample injection, data processing, and analysis. The platform paves the way toward a more fully automated mass spectrometry analysis and ultimately closed-loop laboratory workflows involving automated experimentation and analysis coupled to AI-driven experimentation utilizing cutting-edge analytical instrumentation. AutonoMS documentation is available at https://autonoms.readthedocs.io.

## Introduction

Compared with traditional benchtop experimentation, modern life science laboratories are high-throughput and data-centric discovery platforms. This transformation is largely supported by two pillars: (1) experimental hardware automation and (2) informatic and control software integration. Automated robotic laboratory equipment can increasingly perform labor-intensive physical experimental processes including sample preparation, maintenance, and assay execution.^1−3^ In addition to increasing the quantity and quality of data produced, the use of automated labware also produces metadata audit trails at every step of the experimental process to increase data reusability.^4^ A shift is underway from low-throughput manual laboratory operation toward high-throughput screens generating large quantities of raw data which can only be understood through informatic analysis. This creates a new relationship among the scientist, the benchtop, and software. Experimental platforms that can be run through software calls without human supervision can generate large amounts of high-quality data at lower cost to the human scientist to greatly improve the rate of discovery, especially in screening applications in fields such as drug development and metabolic engineering, in which combing through experimental space is often the rate-limiting factor. Such high-throughput screening platforms almost always rely on an analytical measurement of samples of interest. These instrumental “omics” measurements provide the crucial biochemical readout of the system of interest. Despite the promises of integrated hardware–software automation for life science discovery, there remains a great need for further development of automated analytical platforms at the granular end of the omics scale, namely, proteomics and metabolomics. In these realms, analytical instrumentation often remains manually operated and therefore underutilized. As experimentation becomes increasingly automated and high-throughput, analytical instrumentation must keep pace.

Mass spectrometry (MS) is a valuable and broadly used analytical technique in life sciences. The ability to sensitively and broadly detect the molecular components of biochemistry has made it an essential technique for biomarker discovery, drug development, bioprocess development, and basic discovery.^5−8^ A key driver of the technique’s utility has been the continuous development and refinement of MS instrumentation providing increased sensitivity, resolution, reproducibility, and throughput. However, with these benefits comes a high cost. The vast number of acquisition parameters, diverse instrumentation, and varied applications of MS make it a time-intensive technique requiring multiple iteration cycles and substantial hands-on intervention.

Ion mobility-mass spectrometry (IM-MS) integrates a high-throughput separation dimension with MS detection that offers analyte separation on the basis of ion structure (ion mobility) in addition to standard mass separation in complex samples.^9,10^ The drift tube implementation of ion mobility MS (DTIMS) involves the usage of a uniform field ion mobility drift region to separate ionized molecules prior-to mass-to-charge measurement. Ions exhibit different transit times through the drift tube determined by their size, shape, charge, and instrument acquisition parameters. This measured drift time can then be converted via a first-principles relationship to a collision cross section (CCS) value, which is a function of the molecule’s structural properties.^11^ One of the primary advantages of IM-MS separations is the resolution of isomers on the basis of structure, which in some cases can serve as a replacement to slower liquid chromatography separations.^12,13^ However, despite benefiting analytical peak capacity and structural selectivity, ion mobility introduces further experimental complexity into already labor-intensive MS workflows.

**Figure 1:**
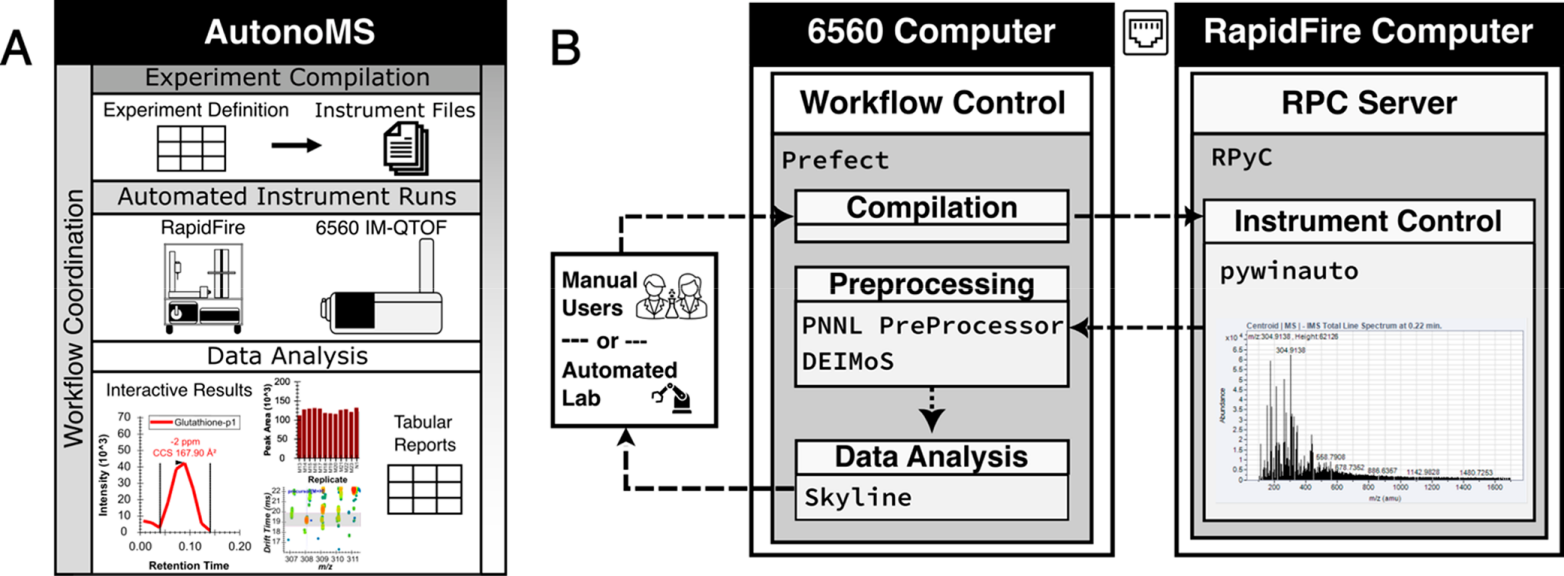
AutonoMS offers walkaway automation of ion mobility mass spectrometry data collection and analysis. (A) AutonoMS integrates software control layers with the Agilent RapidFire–6560 ion mobility mass spectrometry system to provide fully automated data acquisition, raw data handling, data processing, and metabolomic end-to-end analysis, resulting in tabular metabolite reports and interactive Skyline documents. (B) The AutonoMS software stack is hosted on a shared drive between the 6560 and RapidFire control computers. Human laboratory users or an upstream software agent may trigger AutonoMS runs using a tabular experiment definition file. The AutonoMS workflow control is written using Prefect^14^ which coordinates the event-triggered actions of modules responsible for instrument file compilation, instrument control (pywinauto^15^), postacquisition raw data handling including ion mobility demultiplexing and CCS calibration (PNNL PreProcessor^16^ and DEIMoS^17^), and metabolomic data analysis (Skyline^18,19^). Additional modules may be written and incorporated into the workflow to accommodate different instruments or analysis workflows.

**Figure 2:**
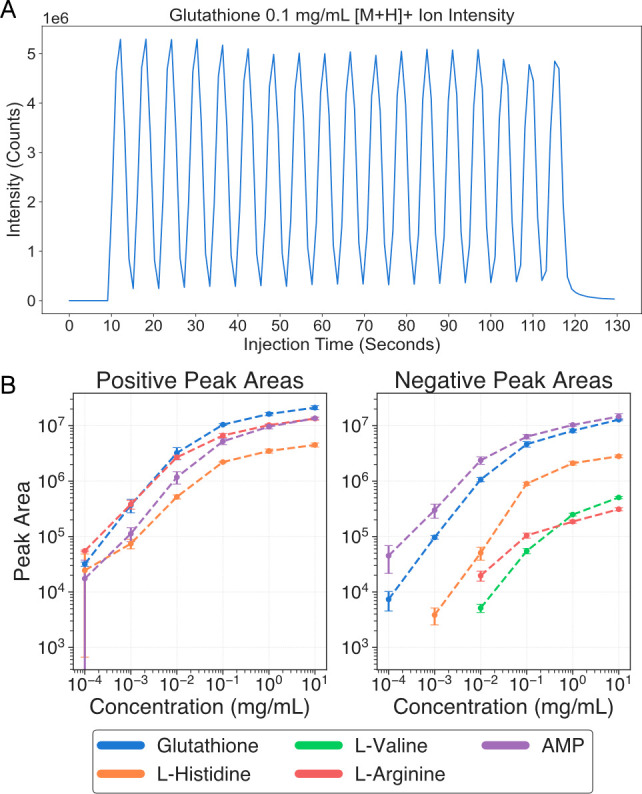
Automated targeted data analysis of the standards with AutonoMS. (A) Detected glutathione [M + H]^+^ ion intensity from an automated AutonoMS analysis of glutathione in 50/50 methanol/water at 0.1 mg/mL injected from separate wells in a robotically dispensed 384 well microplate. (B) Detected peak areas from AutonoMS analysis of robotically prepared triplicate serial dilutions of 5 chemical standards in positive and negative ionization modes robotically dispensed into a 384 well microplate.

**Figure 3:**
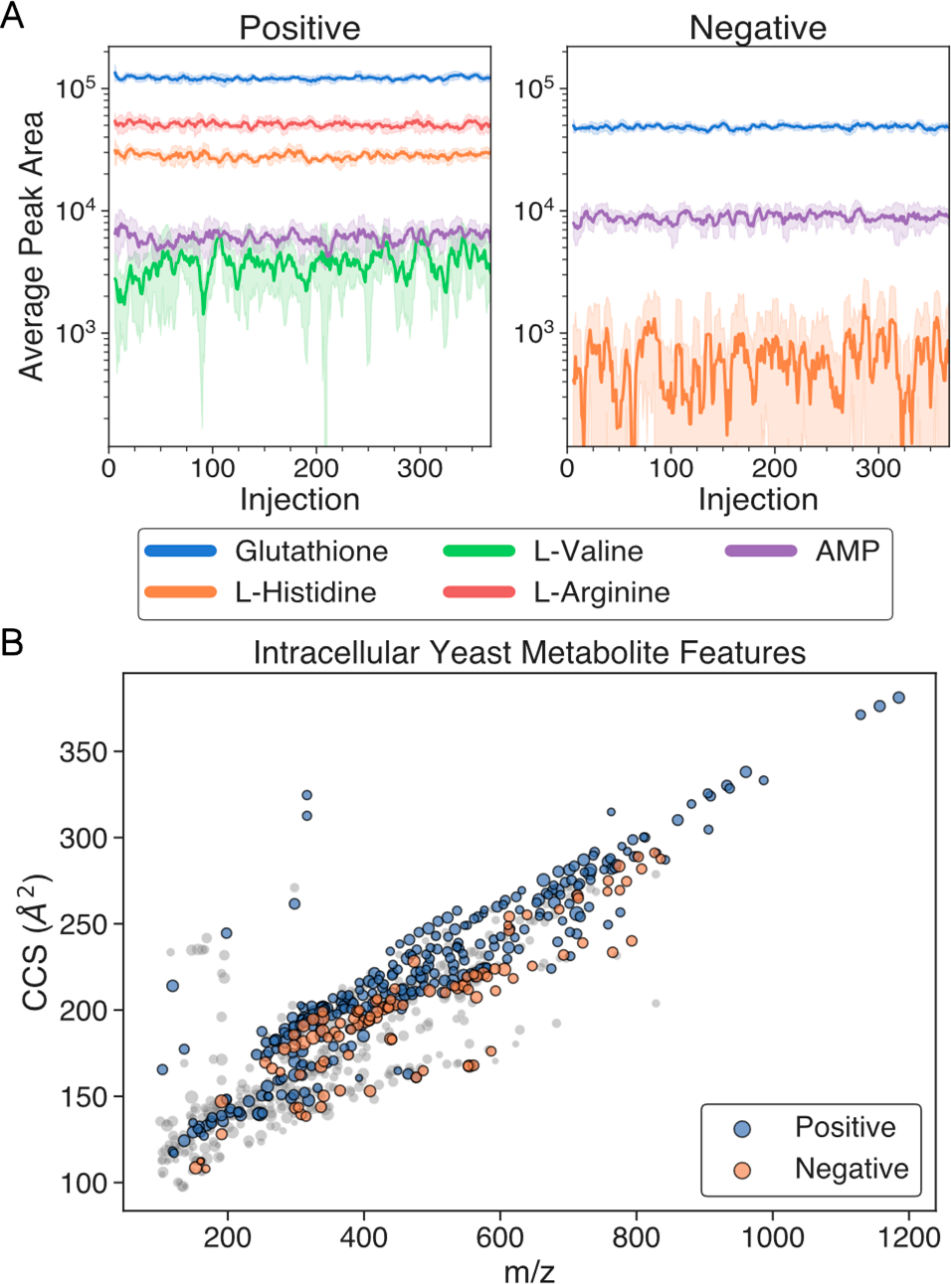
Automated analysis of the extracted intracellular yeast samples with AutonoMS. (A) Detected peak areas in extracted yeast samples across plate injections (368 over 38 min per mode) of the 5 ions used in the chemical standards analysis. Ions correspond to the [M + H]^+^ and [M – H]^−^ adducts in positive and negative modes, respectively. Peak areas shown as the 6-injection moving average (solid lines) together with 6-injection standard deviation (shaded areas). (B) Untargeted metabolite features found across all extracted yeast samples across positive (blue) and negative (orange) ionization modes. Displayed features were present in at least 2/3 of samples in a given mode and had Agilent quality scores greater than 70. A total of 812 features were found, of which 404 involved multiple ions in various ionization states. Single ionization state (*z* = 1) ion features are shown in gray, and marker size is scaled according to log_10_(abundance).

**Figure 4:**
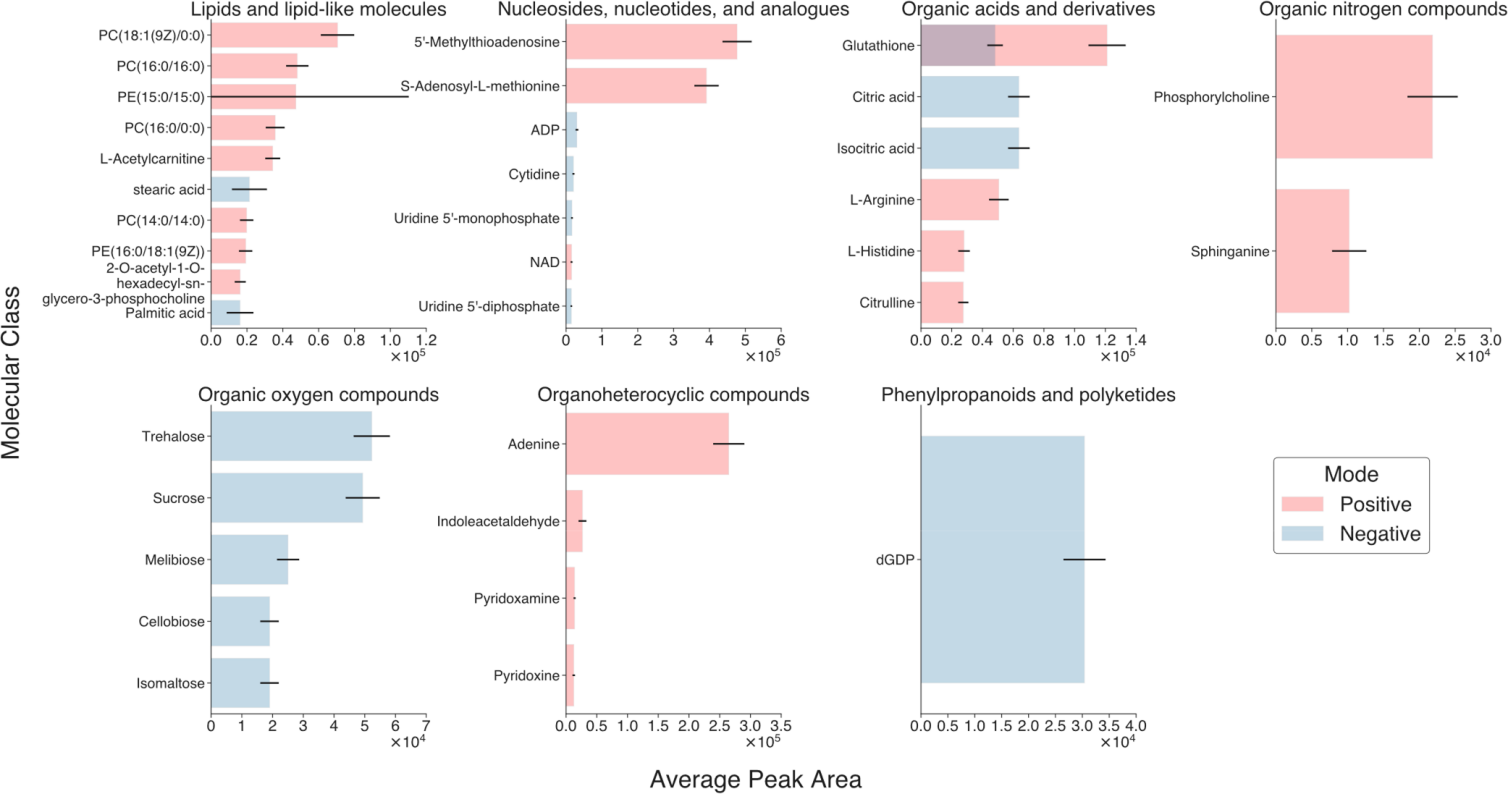
Use of AutonoMS for automated data collection and integration with background knowledge. Panel of 35 metabolites from the Yeast Metabolome Database (YMDB^27,28^) with publicly available collision cross section (CCS) values from the Baker Lab Cross Collision Section Database (CCSDB). Chosen metabolites were detected by the acquisition method described in the Experimental Section and exhibited mean peak areas across extracted yeast injections greater than 10^4^. Metabolites are grouped by their ClassyFire/ChemOnt^32^ superclass labels and are plotted against their mean peak areas across extracted yeast injections per ionization mode. Error bars display the intensity of the standard deviations.

Here we introduce AutonoMS, which offers end-to-end automated runs of MS instrumentation involving sample injection, raw data processing, and metabolomic analysis with little user intervention and is currently written around the Agilent RapidFire^20^ and 6560^21^ DTIMS-QTOF systems (Figure 1A). AutonoMS coordinates instrument control, resource allocation, and data processing across the RapidFire and 6560 control computers using a collection of open-source software libraries (Figure 1B). Sample runs can be automatically triggered from experiment plan files so that either a human user or upstream software agent may design and execute experiments. This means that the AutonoMS platform can be integrated into a larger automated laboratory setting in which software agents control and coordinate multiple experimental, analytical, and informatic modules. Runs may involve multiple acquisition modes, sequences, and variable run parameters. After sample acquisition, data are automatically prepared, processed, and then analyzed via Skyline,^18,19^ producing both interactive results and tabular metabolite summaries. To demonstrate the use of the AutonoMS platform, we analyzed a set of chemical standards chosen from the yeast metabolic network. These standards, serially diluted by an Agilent Bravo liquid handling robot, exhibited the expected dynamic measurement responses as autonomously collected and detected by AutonoMS (Figure 2). We also processed extracted intracellular yeast samples through the platform, indicating its potential utility in automated untargeted and discovery applications (Figure 3 and Figure 4).

## Experimental Section

### Workflow Control

The AutonoMS control flow automation and workflow logic were implemented using Prefect (version 2.10.12).^14^ Prefect is an open-source Python-native workflow manager for orchestrating complex code workflows from modularly defined tasks, smoothly turning Python functions into workflow steps. We chose Prefect because of its open-source nature, active developer community (currently over 200 GitHub contributors), and increasing adoption across industrial data science teams. Its native Python implementation obviates the need to learn a separate workflow domain specific language. The workflow begins by compiling an input tabular experimental definition file into RapidFire XML files. AutonoMS then sequentially triggers an IM-MS acquisition run for each sequence in the experiment definition using the instrument control utilities described below. In our configuration, the workflow currently runs only one sequence at a time in order of appearance in the experiment file. However, the workflow parameters may be modified for laboratories with the capability of running parallel data acquisition on multiple instruments. A sample experiment file is available in the AutonoMS GitHub repository (https://github.com/gkreder/autonoms).

Upon completion of data acquisition for all sequences, the workflow then automatically performs postrun data processing. The RapidFire 365–6560 creates a single Agilent.D raw data file that must be split into .D files corresponding to injections from the individual microplate wells. Currently for RapidFire instruments configured in the BLAZE (direct injection) mode, the RapidFire software can detect injection boundaries but does not correctly split data into the corresponding well files. AutonoMS automatically shifts the file split times according to the detected injection boundaries and assigns them to the correct well plate. A description of the configuration of the RapidFire for the BLAZE mode is provided below.

Data multiplexing is a powerful ion mobility technique for increasing sensitivity.^22^ Our configuration utilizes 4-bit IM multiplexing, but this requires postacquisition data demultiplexing. AutonoMS performs ion mobility data demultiplexing on the individual well files using the PNNL PreProcessor utility.^16^ Before conversion to collision cross section (CCS) values, raw measurements in DTIMS files from the Agilent 6560 must have their drift time values calibrated according to standard measurements with known CCS values. AutonoMS automatically performs this CCS calibration for each injection within a given sequence using the nearest prior injection occurring in the same sequence with sample type “TUNE”. The demultiplexed tune file is converted to mzML^23^ format using the msconvert utility.^24,25^ CCS correction coefficients are then calculated from the mzML tune file using the DEIMoS library for ion mobility data processing^17^ and user-specified reference CCS values. A CCS calibration XML file is generated and copied to each injection .D file in the given sequence. For the experiments outlined in this work, an Agilent ESI tuning mix was used for CCS calibration. A sample sheet containing the Agilent tune ions and their CCS values is available in the AutonoMS GitHub repository. We note that the workflow can be configured to perform this CCS calibration on manually calculated calibration coefficients from separate runs; however, it is recommended to include a tune injection in each sequence for the sake of automation simplicity and data robustness. AutonoMS then performs peak detection and quantification on the demultiplexed CCS calibrated injection files using the Skyline method (described below) via the Skyline Command-Line interface.

### Instrument Control

The pywinauto library (version 0.6.8)^15^ was used to write automation control wrappers around the Agilent MassHunter Workstation Data Acquisition (version 11.0) and RapidFire UI (version 6.1.1.2114) software used to control the 6560 mass spectrometer and RapidFire sampler. Functionalities necessary for automatically running plates according to the user-supplied acquisition method and experimental parameters were implemented. These include reading instrument state, loading files and methods, starting and stopping runs, running the calibrant line, checking the RapidFire vacuum pump pressure, setting run mode, and data file splitting. Of special note is the standard Agilent hardware configuration of separate control desktops for the 6560 and RapidFire connected via ethernet (Figure 1B). To work in this configuration, the codebase is hosted on the network drive shared between the two computers and the AutonoMS workflow is run from the 6560 control computer. A Remote Procedure Call server using the RPyC library (version 5.3.1)^26^ is hosted on the RapidFire computer to execute RapidFire control functions from the 6560 computer. The codebase for RapidFire–6560 instrument control is available in the agilent_methods modules of AutonoMS.

### Data Processing

Peak detection and area quantification were performed using Skyline (version 22.2.0.351).^18,19^ Skyline was chosen because of its open-source nature, ability to handle ion mobility data, and combination of command-line and GUI functionality. Skyline natively supports RapidFire ion mobility data and has previously been used for acquisition workflows such as those described below.^12^ Its command line utilities can be integrated into a fully automated workflow as in AutonoMS and results can later be loaded into the GUI to be verified by the end user. Peak detection was run using the TOF mass analyzer settings at a resolving power of 30 000, an ion mobility resolving power window of 30, and a maximum *m*/*z* of 1700. The full set of Skyline processing parameters in a Skyline document format is available in the AutonoMS repository.

### Metabolite CCS Library

Yeast metabolites were taken from the Yeast Metabolome Database (YMDB)^27,28^ and compared against the Cross Collision Section Database (CCSDB) hosted by the Erin Baker Lab and available at https://brcwebportal.cos.ncsu.edu/baker/. These CCS values were of particular interest, since they were also measured on an Agilent 6560 mass spectrometer for comparison purposes. Metabolites appearing in both YMDB and CCSDB were compiled together with their experimentally observed CCS values for their [M + H]^+^ and [M – H]^−^ adducts. The compiled list of YMDB CCS metabolites in the Skyline transition list format is available in the AutonoMS repository.

### RapidFire Sample Injection

The RapidFire was operated in BLAZE flow injection mode to achieve rapid sample injection and analysis times.^29^ This involves configuring the RapidFire valve tubing such that the sample loop feeds directly into the mass spectrometer outlet rather than through the solid phase extraction (SPE) cartridges. The RapidFire configuration files must also be modified for the valve positions to correctly correspond to sample sipping with this connection configuration. We note that the RapidFire is capable of some automated in-line sample preparation, for example, desalting, via its built-in SPE functionality. This functionality was not utilized for our demonstration experiments since our sample preparation was performed prior to injection. RapidFire BLAZE mode configuration instructions are provided in the Supporting Information. Operating the RapidFire using the SPE functionality via AutonoMS can be done by simply reverting the instrument back to its standard configuration and changing the cartridge and sipper parameters in the input experimental file.

The RapidFire method used involved a sample sipping time of 600 ms followed by 4400 ms of sample elution into the MS. As seen previously,^12^ sipping (aspiration) time is reported rather than injection volume as this is the instrument’s controllable parameter given its mechanism of sample aspiration driven by a vacuum pump. The RapidFire’s sample loop holds roughly 30 μL. This elution time was chosen to ensure baseline peak separation at higher sample concentrations, and we note that this can be reduced for faster cycle times. The mobile phase (pump 1) consisted of 50/50 water/methanol with 0.1% formic acid at a flow rate of 1.25 mL/min. The full RapidFire parameter set is provided in the Supporting Information.

### 6560 Mass Spec Data Acquisition

Mass spectrometry data was collected in IM-QTOF mode with 4-bit multiplexed introduction of the ion packets into the drift tube. Multiplexing has been shown to improve ion utilization and resolving power in IMS;^12^ however, it creates the requirement for additional data postprocessing as described in the workflow management section. The Agilent 6560 was operated in the 100–1700 *m*/*z* range at a frame rate of 1.1 frames/s and a gas temperature of 325 °C. A full description of the QTOF and IM acquisition parameters are available in the Supporting Information.

### Standards Preparation

Dry chemical stocks of glutathione, l-histidine, l-valine, and l-arginine were mixed at room temperature with a stock solution of 50/50 water/methanol to a concentration of 10 mg/mL. These stocks were dispensed into standard 384 well microplates using a Thermo Fisher Combi Multidrop reagent dispenser, and 10-fold serial dilutions were robotically performed using an Agilent Bravo liquid handling robot controlled with the VWorks Automation Control software (version 8.0.0.335, Agilent Technologies).

### Yeast Culturing

*S. cerevisiae* wild-type strain BY4741 (accession number: Y00000) from the EUROSCARF deletant library was revived from −80 °C glycerol stocks by overnight cultivation in YPD media (10 g/L yeast extract, 20 g/L peptone from meat, 20 g/L dextrose) at 30 °C, 220 rpm. The strain was then streaked out on a YPD agar plate and incubated at 30 °C for 3 days. A YPD preculture was inoculated using multiple colonies from the agar plate and then incubated at 30 °C, 220 rpm for 14 h. The cells from the YPD preculture were washed twice (centrifugation at 5000*g*, 5 min) with YNB media (6.7 g/L YNB without amino acids and with ammonium sulfate, 1× amino acid mix, 20 g/L dextrose). Cells were resuspended in 1 mL of YNB media and used as inoculum for the main culture with an initial OD600 of 0.05. The main cultivations were performed in 4 × 250 mL wide-necked baffled shake flasks sealed with cotton stoppers, each with a working volume of 40 mL YNB media. The shake flasks were incubated at 30 °C, 220 rpm. Cultivations were stopped after 24 h postinoculation and the flasks were pooled. Final OD600 was measured to be 3.56 and was used to adjust the 2-propanol alcohol volume in the ensuing extraction method.

### Yeast Quenching and Extraction

Sample preparation and intracellular metabolite extraction followed a previously established protocol.^30,31^ Samples were quickly transferred to 15 mL centrifuge tubes (5 mL per tube) containing absolute methanol (99% purity) prechilled to −80 °C. The ratio between sample and methanol was kept at 1:1 v/v. Tubes were kept in dry ice during the process and were transferred to a centrifuge and spun for 5 min at 3000*g* and −9 °C. The supernatant was then discarded, and the pellet was transferred and stored at −80 °C. Samples were lyophilized (−40 °C, 0.1 mbar) overnight and kept at −80 °C pending extraction. Metabolite extraction was performed by adding 75% 2-propanol (preheated to boiling temperatures, with a ratio of 1 mL of 2-propanol per 1 mg of sample) to the lyophilized yeast biomass in 15 mL centrifuge tubes. Sample weight was estimated from optical density (0.34 mg DCW/mL per 1 OD600). Samples were placed on a heating block for 1 min at 100 °C, then shaken and vortexed for 2 min, followed by an additional 3 min on the heat block. Samples were then cooled for 15 min at 4 °C before centrifuging for 20 min at 3200 g and 4 °C. The supernatant was filtered through a 0.45 μm nylon filter, transferred to 50 mL centrifuge tubes, and stored at −20 °C until analysis. Samples were transferred to a standard 384 well microplate for AutonoMS injection by using a Thermo Fisher Combi Multidrop reagent dispenser.

## Results and Discussion

### Walkaway Automation

Over the course of our experimentation, we found that AutonoMS enabled reliable automated runs and analysis of data from simple experimental definition files without any need for human intervention (Figure 1). The AutonoMS platform integrated smoothly into our automated laboratory workflows and provides an event-triggered software-compatible interface to a larger automated environment. Resources can be scaled automatically through the control workflow in which specific tasks are granted user-defined resource usage and concurrency rights. We found this was crucial for automating this combination of data acquisition, data preprocessing, and data analysis tasks with varying degrees of interdependencies and resource demands. Through the control workflow, the informatic steps of the platform can be run on remote or distributed resources to cut down on computation time. Instrument data acquisition for a given experiment definition runs in its entirety before initiating the informatic portions of the pipeline. As such, multiple sequences can be run from the same microwell plate while minimizing sample evaporation time. The targeted data and untargeted assays described below involved laboratory robotics and multiple data sequences and acquisition modes per experiment. We found that aside from hardware maintenance and physical sample transfer, usage of the AutonoMS platform eliminated the need for human intervention or even presence during the process of metabolomic data acquisition and analysis.

### Targeted Data Analysis of Standards

We tested the use of AutonoMS in a targeted data analysis metabolomics application by automated sampling and analysis of a chemical standard, glutathione, at a set concentration of 1 × 10^–1^ mg/mL in a stock solution of 50/50 methanol/water (Figure 2A). AutonoMS, running the direct injection ion mobility method described in the Experimental Section, enabled the automated analysis of the known standard with an injection time of 5 s per sample with consistent performance. The platform autonomously injected, processed, and detected the [M + H]^+^ adduct in positive ion mode without human intervention. We also tested the AutonoMS platform on robotically prepared triplicate 10-fold serial dilutions of five standards: glutathione, l-histidine, l-valine, l-arginine, and adenosine monophosphate, from a starting concentration of 10 mg/mL (Figure 2B). Peaks areas were filtered to include only detections at levels 5× higher than those in 50/50 water/methanol blanks. The platform similarly autonomously collected data and reproducibly detected the standards with a linear peak area response range in the 10^–4^–10^–1^ mg/mL range. In both cases, no human intervention was required, other than cleaning of the instruments and transfer of the prepared sample microplates to the RapidFire. These panels lead us to conclude that (1) the AutonoMS platform automates existing targeted data analytical workflows and (2) using the automated workflow produces robust and consistent results useful to downstream human users or software processes.

### Metabolomic Fingerprinting of Yeast

To investigate the utility of the AutonoMS platform in systems biology discovery applications, we cultured and prepared yeast samples to study their intercellular metabolomic content via untargeted metabolomic fingerprinting (Figure 3 and Figure 4). Extracted intracellular yeast samples were pooled and dispensed into a standard 384 well microplate after which AutonoMS was used to automatically run whole-plate sequences of injections in both positive and negative ionization modes. 368 consecutive injections were run in each mode (leaving the first plate column reserved for tune ions) for a total injection time of 38 min per mode. Ion behavior in this complex sample matrix largely agreed with exhibited behavior in the targeted data analysis standards test (Figure 3A). Ions with higher intensities had relatively consistent peak areas across the plate injections, with peak area consistency decaying dramatically around the 1 × 10^4^ mark due to poor counting statistics.

The preprocessed data produced by AutonoMS were also run through untargeted feature finding with Mass Profiler (version 10.0.195, Agilent Technologies), yielding 812 metabolite features across positive and negative mode with both (1) a Q-Score greater than 70 and (2) occurrence in at least 2/3 of a given ionization mode’s injections (Figure 3B). Of these features, 404 were higher-quality features involving multiple ions in various ionization states. This Mass Profiler analysis was performed manually but could be automated and integrated into AutonoMS using DEIMoS^17^ if it suits the end-user’s needs. AutonoMS automatically ran these yeast injection data through the Skyline pipeline using the YMDB CCS library (described in the Experimental Section). The reports generated from the AutonoMS runs facilitated compilation of a panel of 35 detectable YMDB intracellular metabolites with publicly available CCS values filtered according to the earlier tests (displaying an average peak area greater than 10^4^ across injections) using the ion mobility direct injection method described in the Experimental Section. These metabolites are shown in Figure 4 grouped by their ClassyFire/ChemOnt^32^ superclass labels together with their mean TIC-normalized peak areas across injections. We note that further automated data collection and analysis modules can be incorporated via AutonoMS to improve the application-specific detection performance of such a workflow. Usage of the AutonoMS platform enabled hands-off automated metabolomic profiling in the context of the existing background knowledge of yeast metabolism. In this case, usage of the platform dramatically decreased the time and effort required to characterize yeast samples and produce interpretable results in the context of this background knowledge. This opens the door toward incorporation of a cutting-edge analytical platform into more fully autonomous discovery applications in which AI software agents control experimentation, interpret results, and run further rounds of experimentation as has been proposed and demonstrated previously.^33−35^ Metabolomics-based biological discovery, especially in yeast, is a rich field. Previous work has demonstrated the utility of applying rigorous mass spectrometry methods toward measurements of yeast metabolites.^36,37^ Integrating these with automated culturing, sampling, additional data modalities, and modeling techniques has yielded powerful approaches.^38,39^ Existing approaches have demonstrated the potential of in-line SPE-IM-MS exometabolome analysis in live cultures.^40^ AutonoMS should facilitate such multifaceted approaches, allowing for granular control logic and flexibility in new techniques. We also note that in addition to downstream discovery, the automated characterization of well-behaved metabolites for a given instrument acquisition method immediately opens the door to closed-loop automated MS acquisition methods development.

## Conclusions

Analytical instrumentation must keep pace with the increasing ability of life science laboratories to quickly produce large quantities of experimental samples. Here we introduced AutonoMS, a platform combining cutting-edge ion mobility mass spectrometry instrumentation with software layers capable of end-to-end instrument control, data processing, and metabolomic analysis. From our findings, we conclude that the resulting configuration can be immediately utilized in laboratories already using the same instrumentation to dramatically improve workload and improve results, especially as related to human burden. Even when using human-compiled experiment definition files, the streamlined AutonoMS workflow produced actionable results in about 1/4 of the time compared to the same tasks using the already-optimized conventional Agilent RapidFire–6560 vendor workflow (25 min compared to an hour for the targeted data analysis standards workflow) with much less manual intervention. We also note the extensibility of this platform, since the software workflow coordinates the actions of modular instrument control and informatic components, each of which can be replaced with a new module fitting a given laboratory’s configuration. For example, AutonoMS could be used for untargeted metabolomic feature discovery or proteomic profiling by swapping out the corresponding Skyline metabolomics module. The current RapidFire–6560 instrumentation setup is already capable of a large range of analyses including proteomics. A new instrument could be controlled by AutonoMS by calling its instrument control wrappers in the workflow. Multiple instruments can be run in parallel simply by modifying the workflow configuration parameters. This process can be made easier through instrument vendor cooperation, particularly with regard to whether high-level users will have access to an application programming interface (API) to allow direct command-line control of all instrument functions rather than requiring all commands to be passed manually to the instrument through a graphical user interface (GUI) by means of interactive mouse-clicks and pull-down menus on a screen. As described in the Experimental Section, instrument control for the RapidFire–6560 required GUI automation. General laboratory automation outside mass spectrometry remains a challenge. For example, our yeast quenching and extraction protocol was performed manually save for the final step of robotically dispensing samples into the injection plate using the Multidrop dispenser given logistical and resource constraints. With the proper automated liquid handling equipment and laboratory space, this protocol can be fully automated, and the capabilities of AutonoMS would allow for completely hands-off analysis of samples from culture to output results. Looking further ahead, we believe AutonoMS opens the door to closed-loop automation utilizing cutting-edge analytical instrumentation in which automated laboratories produce samples, transfer them to the instrument, trigger AutonoMS runs, and then use the processed results to perform the next round of experimentation. For example, a setup combining automated software-controlled chemostats, liquid handling robotics, centrifuges, heat plates, shakers, freezers, and the RapidFire–6560 could be used to culture yeast samples in various conditions, prepare them, and then metabolically analyze them all via software calls with AutonoMS handling the IM-MS portion of the workflow. An AI system sitting on top of this configuration could take produced results and compare them with existing background knowledge to find discrepancies between observed and expected output. Such systems could utilize database systems under development^41^ designed for AI software agent unified access to experimental data, protocol metadata, results, and background knowledge.

## Data Availability

AutonoMS source code, example experimental, configuration, and data processing files are available via GitHub at https://github.com/gkreder/autonoms. AutonoMS experimental run data, manuscript visualization notebooks, and source code are available via Zenodo at 10.5281/zenodo.8318982.

## Supporting Information Available

6560 MS acquisition parameters, RapidFire method parameters, BLAZE mode configuration instructions, and MassProfiler feature finding parameters (PDF)
Video of AutonoMS usage demonstrating a complete run through of the RapidFire–6560 automated workflow on two short sequences, one in positive ionization mode and the other in negative mode (MP4)
