# Supplementary material for: AutonoMS: Automated Ion Mobility Metabolomic Fingerprinting
Source: J Am Soc Mass Spectrom. 2024 Feb 4;35(3):542–50. doi: 10.1021/jasms.3c00396 (PMC10921458; doi:10.1021/jasms.3c00396)
Supplement: Supplementary file 1 — js3c00396_si_001.pdf [file js3c00396_si_001.pdf]

Supporting Information

AutonoMS: Automated ion mobility metabolomic fingerprinting

Gabriel K. Reder[a,b]*, Erik Y. Bjurström[c], Daniel Brunnsåker[a], Filip Kronström[a], Praphapan Lasin[c], Ievgeniia Tiukova[c], Otto I. Savolainen[c,d], James N. Dodds[e], Jody C. May[f], John P. Wikswo[g,h,i,j], John A. McLean[f,g,k], Ross D. King[a,l,m]

[a]Department of Computer Science and Engineering, Chalmers University of Technology, Gothenburg, Sweden 412 96
[b]Department of Applied Physics, SciLifeLab, KTH Royal Institute of Technology, Solna, Sweden 171 21
[c]Department of Life Sciences, Chalmers University of Technology, Gothenburg, Sweden 412 96
[d]Institute of Public Health and Clinical Nutrition, University of Eastern Finland, Kuopio, Finland 702 11
[e]Chemistry Department, The University of North Carolina at Chapel Hill, Chapel Hill, NC USA 27599
[f]Department of Chemistry, Vanderbilt University, TN USA 37235
[g]Vanderbilt Institute for Integrative Biosystems Research and Education, Vanderbilt University, TN USA 37235
[h]Department of Biomedical Engineering, Vanderbilt University, TN USA 37235
[i]Department of Physics and Astronomy, Vanderbilt University, TN USA 37235
[j]Department of Molecular Physiology and Biophysics, Vanderbilt University, TN USA 37240
[k]Center for Innovative Technology, Vanderbilt University, TN USA 37235
[l]Department of Chemical Engineering and Biotechnology, University of Cambridge, UK CB3 0AS
[m]The Alan Turing Institute, UK NW1 2DB

*Email: reder@kth.se

6560 MS ACQUISITION PARAMETERS

Ion Source	Agilent Dual AJS ESI
MS Abs. threshold	200
Ion Mobility Mode	IMS QTOF
Component Model	G6560B
MS Rel. threshold (%)	0.010
Min Range (m/z)	100
Max Range (m/z)	1700
Frame Rate (frames/sec)	1.1
IM Transient Rate (transients/frame)	19
Max Drift Time (ms)	50
Trap Fill Time (μs)	3000.0
Trap Release Time (μs)	100.0
Gas Temp (°C)	325
Gas Flow (l/min)	11
Nebulizer (psig)	45
SheathGas Temp (°C)	275
SheathGas Flow	12
VCap (V)	4000
Nozzle Voltage (V)	1000
Drift Tube Entrance Voltage (V)	(+/-)1800
Drift Tube Exit Voltage (V)	(+/-)224

Table S1. The Agilent 6560 IM-MS acquisition parameters used for the experimental data collection in positive (+) and negative (-) ionization modes.

RAPIDFIRE METHOD PARAMETERS

Note that this method is for Agilent RapidFire BLAZE mode. In BLAZE mode, the Load/Wash cycle corresponds to elution at runtime, so this method consists of 600ms of sample sipping following by 4400ms of sample elution. If the RapidFire is run in normal (non-BLAZE) mode, the cycle names will correspond to the true fluidic action at runtime.

Sipper Height (mm)	1.5
Plate Height	0
Wash Between Sips	FALSE
No. Flushes After Plates	0
Pump 1 Flow Rate (mL/min)	1.25
Pump 2 Flow Rate (mL/min)	0.01
Pump 3 Flow Rate (mL/min)	0.01
Pump 1 [B, C, D] Line %	[50, 50, 0]
Pump 2 [B, C, D] Line %	[100, 0, 0]
Pump 3 [B, C, D] Line %	[100, 0, 0]
Plate Configuration	384-well
Missed Sip Tolerance	10000

Aspirate Cycle Duration (ms)	600
Load/Wash Cycle Duration (ms)	4400
Extra Wash Cycle Duration (ms)	0
Elute Cycle Duration (ms)	0
Reequilibrate Cycle Duration (ms)	0

Table S2. The Agilent RapidFire 365 method parameters used for the experimental data collection. Note that this method was used on an instrument configured in BLAZE mode.

BLAZE MODE CONFIGURATION

Note these instructions are for the RapidFire 365. Minimizing the length of the tubing from the RapidFire to the 6560 will optimize peak sharpness and throughput.

Re-routing fluidic tubing

- Disconnect tubing attached to V1 port 1.

- Disconnect tubing attached to V2 port 2 (which goes to the MS) and connect to V1 port 1.

- Connect the line from V2 port 6 to V2 port 2.

- Keep the intake lines for pumps 2 and 3 submerged in water (solvent doesn't matter, just to keep wet while not in use).

- OPTIONAL. If not using needle washes between samples sips, detach the conduits from the rollers on the front of the peristaltic pump.

Software Configuration Changes

- Make a copy of the RapidFire configuration folder (i.e. the folder chosen at RapidFire UI startup time containing the .cfg configuration files). Give the copied folder a desired name, here referred to as "FIA configs".

- In the FIA configs folder, set MIN_ALLOWABLE_PRESSURE=0.00; in Pump2.cfg and Pump3.cfg.

- In the SampleInterface.cfg file, set the following configuration for the cycle state settings:

STATE1=[0,0,0,600,"Aspirate"];
STATE2=[1,0,0,3000,"Load/Wash"];
STATE3=[1,0,0,0,"Extra Wash"];
STATE4=[1,0,0,6000,"Elute"];
STATE5=[1,0,0,1000,"Reequilibrate"];

- In the ColumnChanger.cfg file, set AUTO_COLUMN_SWITCH=0;

- Make sure to start the RapidFire UI pointing to the correct FIA configs folder when using BLAZE mode and remember to relaunch the UI with the standard configuration folder when operating in normal mode.

UNTARGETED ANALYSIS OF EXTRACTED YEAST

MassProfiler Method Parameters

Measure of Abundance	Max ion volume
Infusion data ion intensity filter	>= 1000.0 count
Isotope model	Common organic molecules
Report single-ion features	TRUE
DT tolerance	±1.5%
Mass tolerance	±(15.0 ppm + 2.0 mDa)
Q-Score	>= 70.0

Table S3. The MassProfiler method parameters used in the untargeted yeast data analysis.
